# The host calcium system contributes to intracellular *Rickettsia* pathogenesis

**DOI:** 10.1128/iai.00363-25

**Published:** 2025-08-21

**Authors:** Jinyi C. Zhu, Jack H. Cook, Mustapha Dahmani, Sean P. Riley

**Affiliations:** 1 Biological Sciences Training Program, University of Maryland College Park 1068, College Park, Maryland, USA; 2 Department of Veterinary Medicine, University of Maryland College Park 1068, College Park, Maryland, USA; 3 Virginia-Maryland College of Veterinary Medicine 212460 , College Park, Maryland, USA; University of California Davis, Davis, California, USA

**Keywords:** *Rickettsia*, calcium, host-pathogen interactions, obligate intracellular bacteria, host-targeted therapeutics

## Abstract

Bacteria in the genus *Rickettsia* are obligate intracellular parasites of the eukaryotic cytoplasm. Pathogenic *Rickettsia* species are exquisitely evolved to only proliferate within eukaryotic host cells, particularly within endothelial cells of the mammalian vasculature. Through evolution in this very specific niche, *Rickettsia* have developed an inextricable dependence on multiple host functions. This absolute dependence on host cell biology offers a potential strategy for antibacterial development called host-targeted therapeutics. A previous screen of compounds that specifically target mammalian cell biology indicated that host-targeted calcium channel blockers (CCBs) inhibit *Rickettsia conorii* proliferation within human cells. CCBs are routinely prescribed to human patients as antihypertensives or antianginals that function by disrupting the calcium ion equilibrium in vesicula/cardiac smooth muscle cells. To further investigate the potential anti-*Rickettsia* activities of CCBs, we sought to define the interaction between pathogenic *Rickettsia* and the host Ca^2+^ system. Achieved data demonstrate that CCBs inhibit *Rickettsia* proliferation within endothelial cells, and that physical disruption of the host Ca^2+^ ion gradient also disrupts *Rickettsia* growth. Additional analyses reveal that *Rickettsia* infection leads to a rapid and persistent disruption of the host Ca^2+^ equilibrium. By querying *Rickettsia* pathogenesis, we demonstrate that some CCBs marginally disrupt rickettsial adherence to the host cell or induce apoptosis. However, all tested CCBs universally and significantly disrupt the ability of *Rickettsia* to polymerize actin. Together, these data demonstrate that CCBs possess anti-*Rickettsia* properties that function by disrupting rickettsial actin polymerization, and these results highlight the complex interdependence of *Rickettsia* and host cell biology.

## INTRODUCTION

Human Rickettsioses are potentially fatal diseases caused by infection with obligate intracellular bacteria of the genus *Rickettsia*. These infections have claimed millions of lives during multiple historical outbreaks (1–4). Contemporary infections like Rocky Mountain spotted fever caused by *Rickettsia rickettsii*, Mediterranean Spotted Fever caused by *Rickettsia conorii*, and *Rickettsia parkeri* rickettsiosis are on the rise, with many other rickettsial diseases found throughout the world (3–9). Current case fatality rates for severe human *Rickettsia* infections range from 5% to 10% (5, 6, 9). However, delayed treatment drastically increases the case fatality rate to 40%–50% among those patients who do not receive treatment before 8 days after symptom onset (9). While pathogenic *Rickettsia* species have been well-documented in the USA since the early 1900s (10–12), the public health impact of *Rickettsia* is expanding due to the detection of new *Rickettsia* pathogens as well as climate- and globalization-driven changes to arthropod host range (7, 9, 13, 14).

*Rickettsia* are obligate intracellular bacteria that have evolved to rely on the host cell for many functions that are normally considered essential for life (15). These bacteria invade nucleated cells by adhering to the host cell surface receptors (16–22). This binding initiates a cascade of host signaling events to induce phagocytosis of the bacilli (23, 24). Once inside the host, the bacteria rapidly escape the nascent phagosome to establish a cytoplasmic intracellular replicative niche (22, 25). While in the host cytoplasm, *Rickettsia* employ a variety of strategies to modulate host cell biology, including stealing host metabolites (15), inducing actin polymerization (25–27), and modulating host apoptosis (28–31). Conversely, under certain conditions, the host cell can detect the pathogen and respond by eliciting innate immune responses (31–34). Thus, there is an elaborate “tug-of-war” that occurs between cytoplasmic *Rickettsia* and host cells.

*Rickettsia* are intrinsically resistant to the vast majority of available antibiotics (9, 35–39). Doxycycline is the only FDA-approved treatment in the USA, leaving a critical lack of treatment options for rickettsial diseases (9). Accordingly, some researchers have sought to identify alternative treatments for Rickettsioses, including exploring host-directed therapeutic approaches (40–44). The underlying logic for these studies is: since *Rickettsia* are obligate intracellular parasites, exogenous modulation of the host environment through bioactive chemicals may create a hostile intracellular environment for *Rickettsia* proliferation (42, 44–46). A previous screen performed by Czyz et al. pinpointed the potential of calcium channel blockers (CCBs) to prevent intracellular proliferation of the intracellular pathogens *Coxiella burnetii*, *Brucella abortus*, *Legionella pneumophila,* and *Rickettsia conorii*, suggesting the potential of CCBs as novel anti-intracellular antibacterials. These findings prompted us to investigate the role of Ca^2+^ during *Rickettsia* infection of mammalian endothelial cells.

The calcium ion, Ca^2+^, is the most abundant metal ion in the human body, and proper distribution of this ion is vital for cellular homeostasis and signaling (for review, see references 47, 48). Broadly speaking, eukaryotic cells employ Ca^2+^-ATPases and Na^+^/Ca^2+^ exchangers to maintain a cytoplasmic Ca^2+^ concentration that is 10,000–20,000 times lower than the corresponding extracellular concentration (47, 48). The metabolic cost associated with maintaining this calcium gradient benefits the eukaryotic cell by supplying a preassembled molecular signal, whereby gated Ca^2+^ channels control ion influx to feed an exceptionally powerful cytoplasmic sensing system (48, 49). This calcium signaling system includes many calcium-binding proteins that relay fluctuations in cytoplasmic Ca^2+^ to signaling cascades (47, 49–52). CCBs are a group of pharmaceuticals that interfere with voltage-gated Ca^2+^ channels to obstruct Ca^2+^ influx, with the eventual result being reduced hypertension (53, 54). Given the known bioactive targets of CCB efficacy, we infer that disruption of host Ca^2+^ homeostasis may also create an inhospitable cytoplasmic environment for *Rickettsia* proliferation.

Many calcium-regulated mammalian signaling pathways facilitate pathogenesis of intracellular bacteria, including (i) internalization of *Listeria monocytogenes* and *Shigella flexneri *(55–57); (ii) phagosomal escape of *L. monocytogenes* and *Mycobacterium tuberculosis *(52, 58, 59); and (iii) modulation of immune response to *Helicobacter pylori* (52, 60). In addition to these more distantly related bacteria, *Ehrlichia* spp. and *Orientia tsutsugamushi* manipulate the host calcium gradient (61–65). Due to the importance of Ca^2+^ in cellular homeostasis and signaling, deciphering how calcium is manipulated during *Rickettsia* infection could inspire further therapeutic development against multiple other intracellular pathogens (66, 67).

The overarching goals of this project were to understand how calcium influences the *Rickettsia*-mammal interaction and to examine the potential for CCBs as a treatment for Rickettsiosis. Our findings help bridge the gaps in our knowledge about *Rickettsia*-host manipulation tactics and advance our understanding of mammalian calcium physiology.

## RESULTS

### Disruption of host calcium equilibrium reduces *Rickettsia* proliferation

A previously performed study identified CCBs as bioactive host-targeted compounds that restrict intracellular proliferation of multiple bacteria, including *Rickettsia conorii,* within THP-1 macrophage-like cells (40). With the hypothesis that CCBs are anti-*Rickettsia* compounds that function by modulating the host cell, we initially sought to estimate the antibacterial activity of three diverse CCBs against *R. conorii* when propagated in EA.hy926 endothelial cells (68). We employed three different CCBs in our studies: (i) Bepridil is a diamine with broad calcium channel antagonism but no structural similarity to other classes of CCBs (69). (ii) Verapamil is a widely used first-generation phenylalkylamine (70). (iii) Nisoldipine is a dihydropyridine that is chemically related to the majority of other CCBs (71). In addition, some analyses throughout this manuscript required live-cell analysis. Thus, we were restricted to the biosafety level-2 (BSL-2) species *Rickettsia parkeri* for some analyses, but we aimed to perform analyses using the more clinically relevant BSL-3 species *R. conorii* whenever the samples could be inactivated prior to analysis.

To first examine the antibacterial properties of CCBs, endothelial cell EA.hy926 monolayers were pretreated with increasing concentrations of Bepridil, Verapamil, and Nisoldipine prior to *R. conorii* infection at a multiplicity of infection (MOI) of 3 bacteria per host cell. This MOI was chosen because it consistently resulted in the highest quantity of *Rickettsia* after 3 days of growth without infection-induced toxicity to the host cells. After 3 days of bacterial proliferation at 37°C 5% CO_2_, the cultures were fixed, permeabilized, stained with a fluorescent anti-*Rickettsia* antibody, and analyzed in a plate reader. The quantity of *Rickettsia* present in each sample was equal to the total fluorescence. Data were transformed using controls, including mock-treated *R. conorii-*infected wells as 100% *Rickettsia* quantity and mock-infected wells as 0% *Rickettsia* quantity. Since reduction in the quantity of *Rickettsia* could stem from either antibacterial effects or toxicity to the host cell, we also determined the inherent toxicity of each CCB using the common MTT assay with the controls: mock-treated EAhy.926 cells as 100% host survival and dimethyl sulfoxide (DMSO)-killed EAhy.926 cells as 0% host survival. Each CCB concentration was analyzed in triplicate, and controls were performed in sextuplicate.

**Fig 1:**
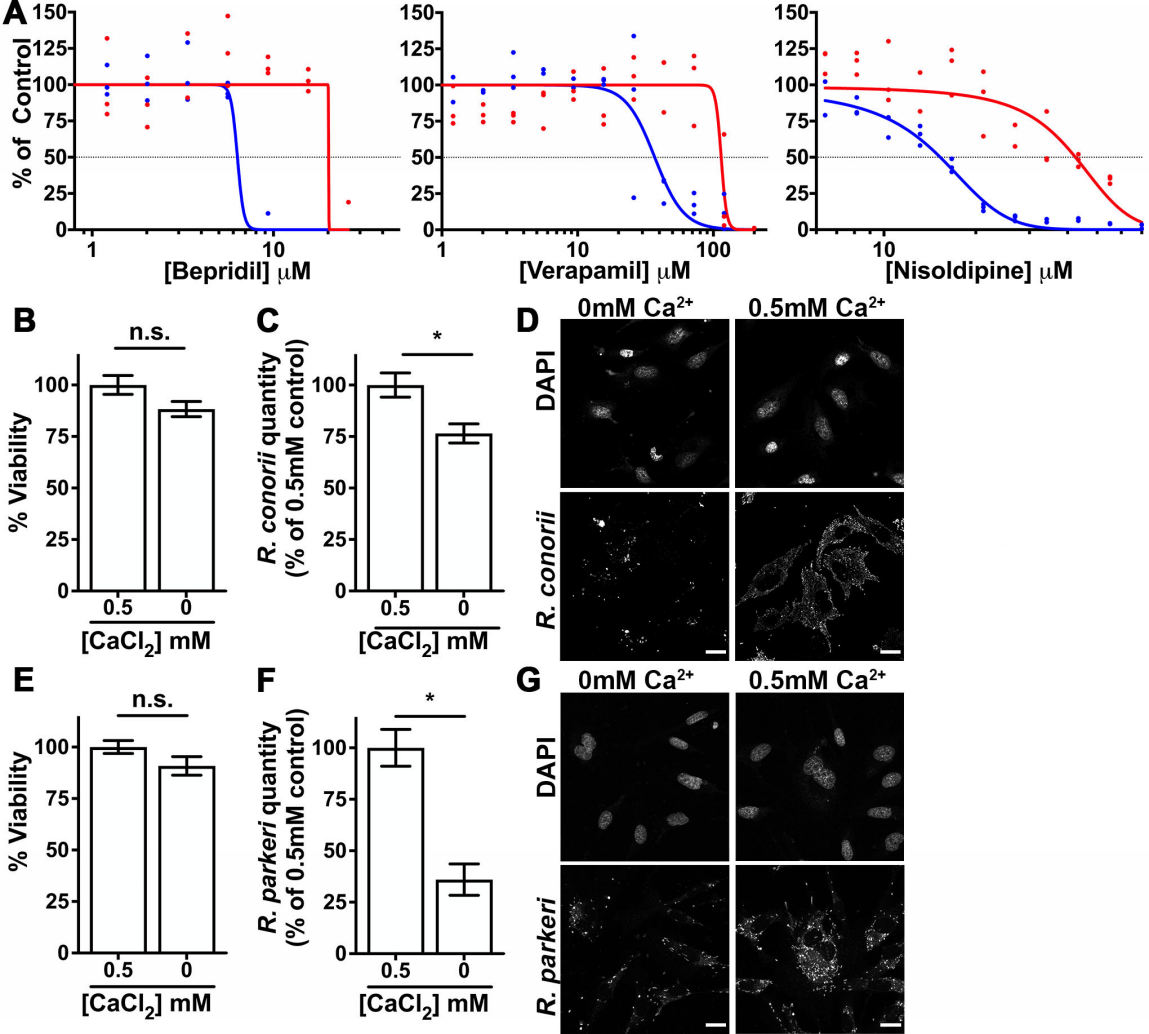
The host Ca^2+^ gradient contributes to *Rickettsia* growth. (**A**) Effect of the calcium channel blockers Bepridil, Verapamil, and Nisoldipine on *R. conorii* growth (blue line) and toxicity to EA.hy926 cells (red line) after 3 days of culture. *Rickettsia* quantities are transformed to 100% (mock-treated) and 0% (mock-treated uninfected). Uninfected host cell survival is transformed to 100% (mock-treated) and 0% (DMSO-killed) survival. Lines are variable slope nonlinear fits. (**B**) Viability of EA.hy926 cells after 3 days in Ca-free media or Ca-free media supplemented with 0.5 mM CaCl_2_. n.s. *P* > 0.05 by Student’s *t*-test. (**C**) Quantity of *R. conorii* after 3 days of growth within EA.hy926 cells in the same media. *P < 0.05 by Student’s *t*-test. (**D**) Immunofluorescence micrograph of host nuclei and *R. conorii* after 3 days of infection in the same media. Scale bar = 20 µm. (**E**) Viability of EA.hy926 cells after 3 days in Ca-free media and Ca-free media supplemented with 0.5 mM CaCl_2_. n.s. *P* > 0.05 by Student’s *t*-test. (**F**) Quantity of *R. parkeri* after 3 days of growth within EA.hy926 cells in the same media. (**G**) Immunofluorescence micrograph of host nuclei and *R. parkeri* after 3 days of infection in the same media. Scale bar = 20 µm.

As shown in Fig. 1A, blue lines, by applying a variable slope linear regression to the quantity of *Rickettsia* in each well, we determined that each drug had a different 50% tissue culture inhibitory concentration (TCIC_50_) for *Rickettsia* growth. The observed TCIC_50_ for Bepridil is 6 µM, Verapamil is 37 µM, and Nisoldipine is 15 µM. Similarly, each CCB has inherent toxicity to the EA.hy926 cells with 50% tissue culture toxicity (TCTox_50_) of 20 µM for Bepridil, 144 µM for Verapamil, and 42 µM for Nisoldipine (Fig. 1A, red lines). Since inhibition of *Rickettsia* growth occurred at substantially lower concentrations than toxicity to the host cell, we conclude that all three CCBs inhibit *R. conorii* propagation at non-toxic concentrations, and that these compounds possess *bona fide* anti-*Rickettsia* activity.

To further examine if the host cell Ca^2+^ gradient contributes to *Rickettsia* intracellular growth, we sought to eliminate the extracellular source of Ca^2+^ ions. To this end, we constructed media consisting of Ca^2+^-free JMEM supplemented with dialyzed serum lacking Ca^2+^ and a similar medium where we reintroduced 0.5 mM CaCl_2_. First, to determine if the elimination of extracellular Ca^2+^ was toxic to mammalian cells, the viability of a confluent monolayer of EA.hy926 cells was determined after 3 days in Ca-free media and Ca-free media supplemented with 0.5 mM CaCl_2_. As shown in Fig. 1B, depletion of extracellular Ca^2+^ did not affect EA.hy926 cells’ fitness. We next infected the EA.hy926 with *R. conorii* for 3 days. As shown in Fig. 1C, removal of extracellular Ca^2+^ significantly decreased *R. conorii* proliferation in Ca^2+^-free cells as compared to media supplemented with 0.5 mM CaCl_2_. To further corroborate this finding, we visualized similarly treated cells by immunofluorescence microscopy. As shown in Fig. 1D, there were fewer *R. conorii* present in the Ca^2+^-free media despite the presence of similar numbers of host cell nuclei. To validate this finding in other *Rickettsia*, we performed the same analyses using *R. parkeri*. The same phenotypes were observed for *R. parkeri*-infected EAhy.926 cells (Fig. 1E through G). Together, the data documented in Fig. 1 demonstrate that both chemical and physical disruption of the Ca^2+^ gradient are detrimental to *Rickettsia* growth.

### *Rickettsia* infection disrupts the calcium gradient of the host cell

**Fig 2:**
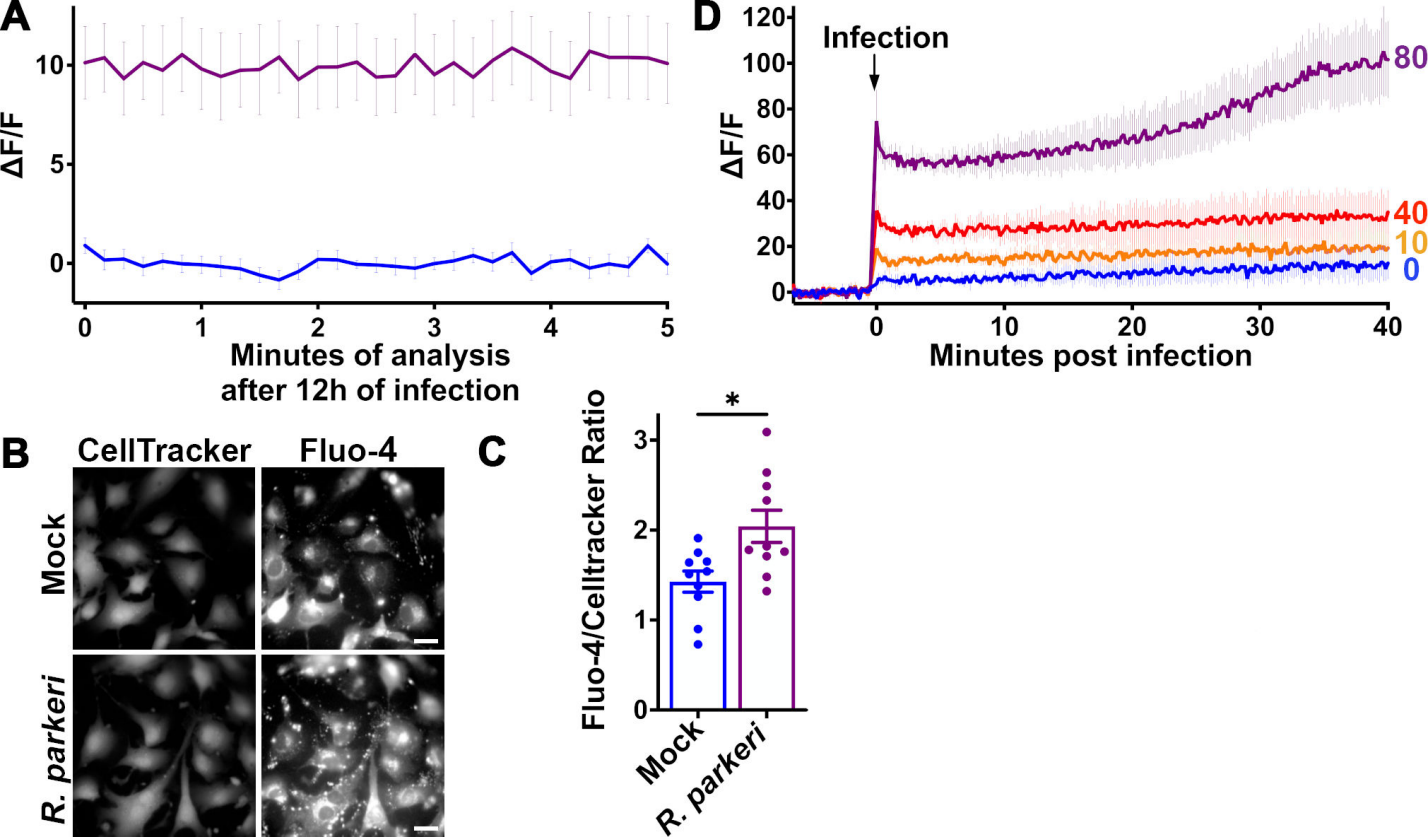
*Rickettsia* infection stimulates an increase in host cytoplasmic calcium concentration. (**A**) Analysis of host internal Ca^2+^ ([Ca2^+^]i) using the calcium-dependent fluorescent indicator Fluo-4, with the data expressed as change in fluorescence over baseline fluorescence ($\Delta F/F=\frac{F_{timepoint}-F_{average\;baseline}}{F_{average\;baseline}}\times 100$). [Ca^2+^]i was analyzed after 12 hours of MOI = 10 *R*. *parkeri* infection of EA.hy926 cells (purple) as compared to mock-infected control (blue). (**B**) Live cell microscopic imaging of EA.hy926 cells stained with Fluo-4 and Cell Tracker Blue after 12 hours of MOI = 10 *R*. *parkeri* infection or mock treatment. Scale bar = 20 µm. (**C**) Ratio of Fluo-4 to CellTracker Blue fluorescence in 10 different microscopic fields. *P < 0.05 by Student’s *t*-test. (**D**) Initial infection of EA.hy926 cells with different MOI *R. parkeri* produces a rapid increase in [Ca^2+^]i as compared to mock-infected cells (blue). This immediate calcium influx is followed by a secondary gradual accumulation of [Ca^2+^]i.

The anti-*Rickettsia* activity of CCBs prompted us to explore two different hypotheses. First, *Rickettsia* infection disrupts the normal host cell Ca^2+^ equilibrium, and second, *Rickettsia* hijacks the host Ca^2+^ system to its own benefit. To first assess *Rickettsia*-induced changes to intracellular calcium concentration ([Ca^2+^]i) within the host cell, we used the cytoplasmic calcium indicator Fluo-4 AM. Since this is a live analysis, we were limited to the relevant biosafety level 2 *Rickettsia* species *R. parkeri*. We first examined [Ca^2+^]i after 12 hours of infection at MOI = 10. This time point was chosen as it is generally understood as the time before *Rickettsia* starts replicating (25, 72). As shown in Fig. 2A, the 12 hour *R*. *parkeri*-infected cells demonstrate elevated [Ca^2+^]i as compared to mock-infected cells. To further substantiate this observation, 12 hour *R*. *parkeri*-infected EA.hy926 cells were again loaded with Fluo-4 but also with the fluorescent cell stain Cell Tracker Blue and observed by immunofluorescence microscopy. As shown in Fig. 2B, both *R. parkeri*- and mock-infected cells were visible with Cell Tracker Blue; however, the *R. parkeri*-infected cells demonstrated elevated Fluo-4 fluorescence. Micrographs were subsequently analyzed to determine the ratio of Fluo-4 and Cell Tracker Blue fluorescence intensity. *R. parkeri*-infected cells demonstrate an increased Fluo-4: Cell Tracker Blue ratio as compared to uninfected controls, indicating that infected cells have a higher [Ca^2+^]i (Fig. 2C).

To further define the temporal relationship between *Rickettsia* infection and [Ca^2+^]i, we next analyzed changes to [Ca^2+^]i immediately after infection. EA.hy926 cells were loaded with Fluo-4 as above, but infected with MOI = 0, 10, 40, or 80 *R*. *parkeri*. As shown in Fig. 2D, there is a clear and rapid dose-dependent increase in cytoplasmic [Ca^2+^]i, indicating that there is a direct correlation between *Rickettsia* quantity and degree of Ca^2+^ influx into the host cytoplasm. Since this increased intracellular Ca^2+^ is likely detected by a plethora of cytoplasmic Ca^2+^ sensory proteins (47, 49), *Rickettsia* infection likely changes the physiology of the host cell in a cytoplasmic Ca^2+^-dependent manner. Together, these results reveal that *R. parkeri* infection induces a rapid and persistent disruption of the host Ca^2+^ gradient.

### Two CCBs reduce the association of *R. parkeri* with the host but do not influence invasion

**Fig 3:**
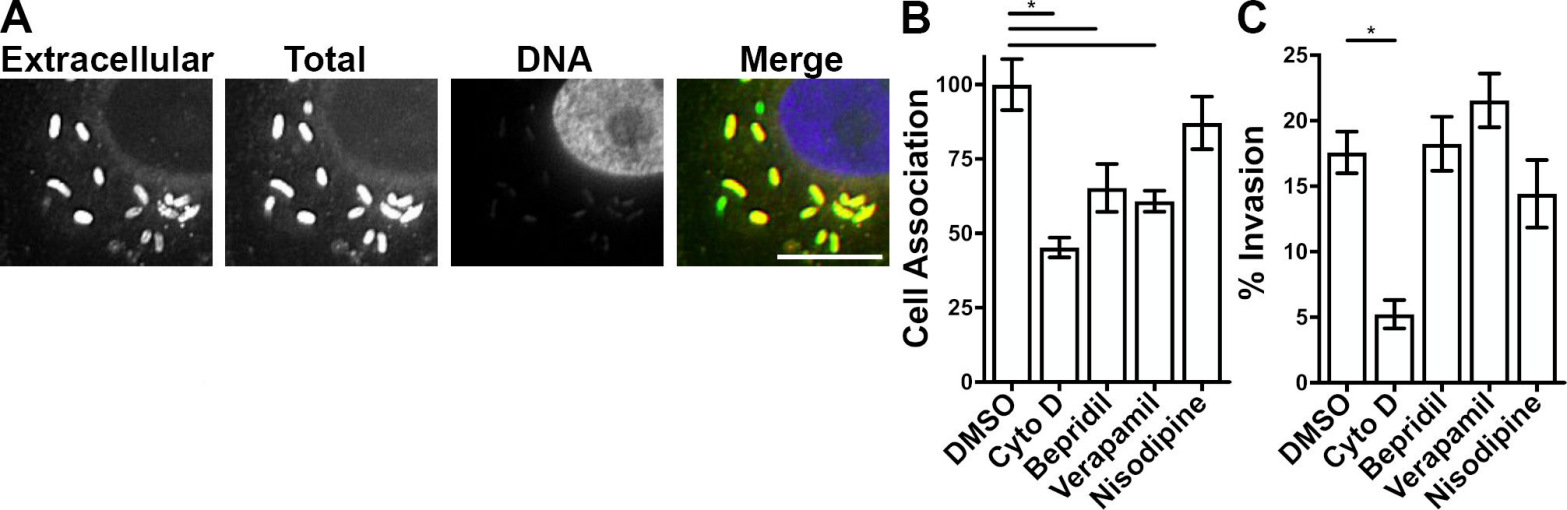
Some CCBs modulate *Rickettsia* cell association but not invasion. (**A**) Representative example of a micrograph to identify extracellular bacilli, total bacilli, and host nuclei with merged image colored as red, green, and blue, respectively. Note the single-colored green bacilli that have invaded the host cell. Scale bar = 5 µm. (**B**) Frequency of *R. parkeri* association with EA.hy926 cells treated for 30 minutes with noted drugs and expressed as % of DMSO-treated control. *P* < 0.05 by one-way analysis of variance (ANOVA) with Dunnett’s multiple comparisons test. (**C**) Frequency of *R. parkeri* invasion into EA.hy926 cells treated for 30 minutes with noted drugs and expressed as % of DMSO-treated control. *P* < 0.05 by one-way ANOVA with Dunnett’s multiple comparisons test.

Having obtained data supporting the concept that disruption of the Ca^2+^ equilibrium generates an inhospitable host environment for bacterial growth (Fig. 1) and that *Rickettsia* infection disrupts the host cell Ca^2+^ equilibrium (Fig. 2), we next sought to determine what stage(s) of *Rickettsia* pathogenesis require a fully functioning Ca^2+^ equilibrium/system. A previous finding hinted at the potential effect of a divalent cation ionophore on *Rickettsia* adherence and invasion (73). Therefore, we first examined whether CCBs modulate rickettsial association with or subsequent invasion of host cells. Confluent monolayers of EA.hy926 cells were pre-treated with previously calculated effective concentrations of Bepridil, Verapamil, and Nisoldipine infected with *R. parkeri* (Fig. 1A). After 30 minutes of infection, the samples were fixed, and extracellular *Rickettsia* were labeled using an anti-*Rickettsia* antibody and Alexafluor546-linked secondary antibody. The samples were subsequently permeabilized, and all *Rickettsia* were labeled with an anti-*Rickettsia* antibody and Alexafluor488-linked secondary antibody. This protocol results in double labeling of extracellular bacteria and single labeling of bacteria that have invaded the host cell (Fig. 3A). To quantify *Rickettsia* associated with host cells, the total number of bacteria was divided by the number of nuclei and expressed as a percentage of mock-treated controls. As shown in Fig. 3B, the CCBs Bepridil and Verapamil moderately reduced rickettsial association with the host cell, but another CCB, Nisoldipine, failed to influence *R. parkeri* adherence. We next quantified the rate of rickettsial invasion into host cells by calculating the ratio of single-labeled (intracellular) bacilli versus total bacteria. For this analysis, the potent actin polymerization inhibitor Cytochalasin D was used as a control (19, 74). As shown in Fig. 3C, none of the CCBs significantly changed the rate of rickettsial invasion into the host cell. Altogether, the data in Fig. 3 demonstrate that treatment with two of the CCBs can moderately decrease *R. parkeri* adherence to the host cell, but none of the CCBs influence the rate of invasion.

### Only Verapamil influences apoptosis in *Rickettsia*-infected cells

**Fig 4:**
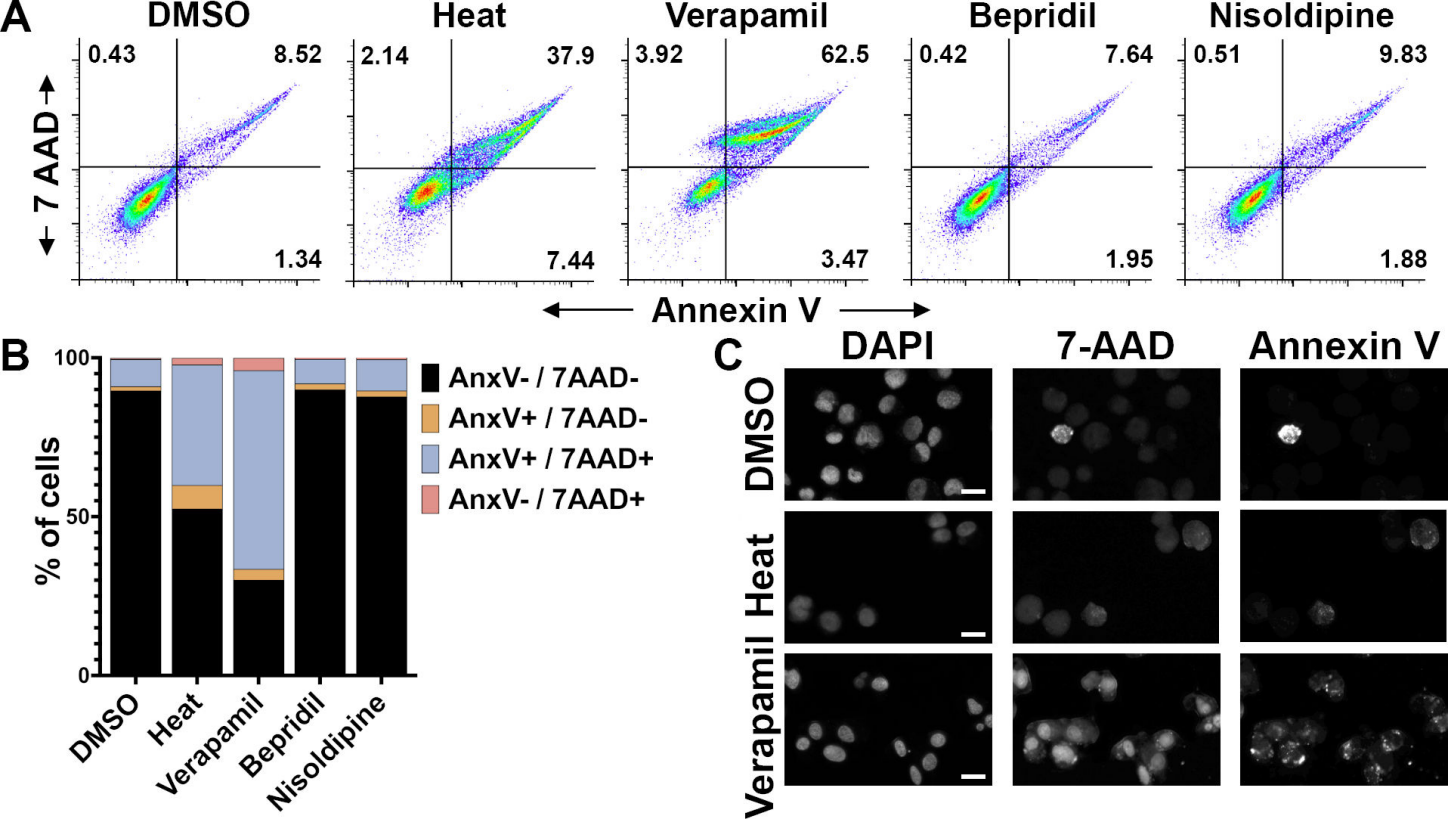
Verapamil induces apoptosis of *R. conorii*-infected cells. (**A**) Flow cytometric analysis of 48 hour *R*. *conorii*-infected EA.hy926 endothelial cells after mock (DMSO), heat shock, or CCB treatment. Cells were labeled with Annexin V to detect apoptotic cells and 7-AAD to detect non-viable cells. Both heat shock and Verapamil treatment increase the frequency of apoptotic or non-viable cells, indicating that these treatments induce apoptosis of infected EA.hy926 cells. (**B**) Frequency of events in each quadrant of the flow cytometric density plots. (**C**) Fluorescence micrographic analysis of *R. conorii*-infected EA.hy926 endothelial cells after mock, heat shock, or Verapamil treatment. Stains include the membrane-permeable DNA stain DAPI, the membrane-impermeable DNA stain 7-AAD, and the apoptosis marker Annexin V. Verapamil treatment of infected EA.hy926 cells increased both 7-AAD and Annexin V fluorescence. Scale bar = 20 µm.

To further define the stage(s) of *Rickettsia* pathogenesis that are influenced by CCBs, we examined the influence of CCBs on rickettsial inhibition of host cell apoptosis (28–30, 75, 76). To this end, EA.hy926 cells were infected with *R. conorii* for 24 hours to establish infection. After 24 hours of infection, the CCBs were added, and the infection was allowed to proceed for an additional 24 hours. Mock-treated cells were used for baseline host cell death, and 55°C/20 minutes heat-shocked cells were used as elevated death controls. Of note, *R. parkeri* strain “Portsmouth” induced high levels of apoptosis, so that species was not used in these analyses (Fig. S1). After 48 hours of total infection, cells were gently liberated from the flask and stained with fluorescent markers of apoptosis, then fixed. Annexin V-PE is an early apoptosis marker of eukaryotic membrane lipid changes, and 7-AAD is a non-permeable DNA stain used to identify non-viable cells. As shown in Fig. 4A, heat shock increased the frequency of Annexin V^+^/7-AAD^+^ cells, indicating that this treatment induces the death of *R. conorii*-infected EA.hy926 cells. Interestingly, Verapamil treatment also increased the frequency of apoptotic/non-viable cells, while the CCBs Bepridil and Nisoldipine failed to increase apoptosis. These changes are apparent in Fig. 4B, where the frequency of events in each quadrant is noted. Verapamil treatment induces death of *R. conorii*-infected cells. To further clarify this finding, we again infected EA.hy926 cells with *R. conorii* for 24 hours prior to treatment. After 24 additional hours, the cells were stained with 7-AAD/Annexin V-PE, fixed with paraformaldehyde, stained with the membrane-permeable DNA dye DAPI, and examined by microscopy. As shown in Fig. 4C, all treatments contained similar numbers of cells as visualized by DAPI staining. However, Verapamil treatment dramatically increased the frequency and intensity of 7-AAD and Annexin V fluorescence. Together, these data demonstrate that only Verapamil induces apoptosis of *R. conorii*-infected cells, but other CCBs do not exert anti-*Rickettsia* effects by inducing apoptosis.

### CCBs all decrease the frequency of *Rickettsia* actin tail formation

**Fig 5:**
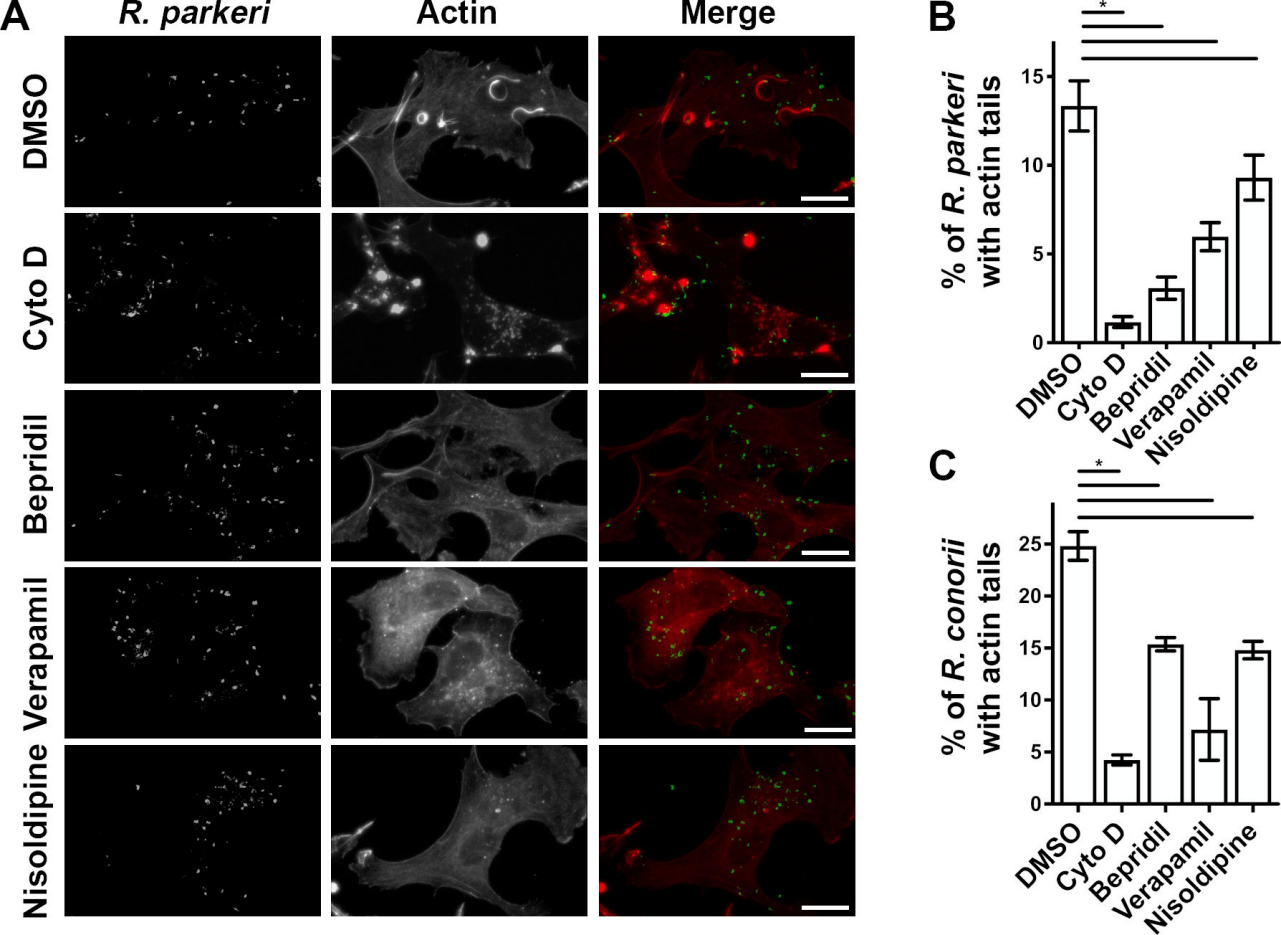
CCB disruption of actin-based mobility. (**A**) Fluorescence micrographs of *R. conorii* and host actin after 48 hours of infection with noted compounds added for the last 24 hours of infection. *R. conorii* (green) containing actin tails (red) are readily observed in mock (DMSO) treated cells, but the frequency of actin polymerization is reduced in CCB and Cytochalasin D treated cells. (**B**) Quantification of *R. parkeri* with observable actin tails after 48 hours of infection of EA.hy926 cells with noted compounds. Treatment with all three CCBs and the actin polymerization inhibitor Cytochalasin D all significantly reduced rickettsial actin polymerization. (**C**) Similar analysis of actin polymerization in *R. conorii*-infected EA.hy926 cells. Scale bar = 20 µm. *P < 0.05 by one-way ANOVA with Dunnett’s multiple comparisons test.

We next sought to determine if CCB treatment modulates rickettsial actin polymerization. Spotted Fever group *Rickettsia* express two surface proteins that polymerize actin to form the idiosyncratic actin tails (77, 78). These actin tails become both more frequent and longer after 24 hours of infection (26, 78, 79). Thus, to assess changes to actin tail formation, EA.hy926 cells were infected for 24 hours before adding CCBs and allowing the infection to proceed for 24 additional hours for a total of 48 hours of infection. As shown in Fig. 5A, rickettsial actin polymerization is readily observable in mock (DMSO)-treated cells when stained with fluorophore-conjugated anti-*Rickettsia* antibody and the actin stain Phalloidin-TRITC. As a control, actin tails are ablated by treatment with the actin polymerization inhibitor Cytochalasin D. Interestingly, treatment with the CCBs Bepridil, Verapamil, or Nisoldipine drastically reduced the frequency of rickettsial actin polymerization. We quantified changes in the frequency of actin tails by analyzing at least 60 cells per treatment to assess the frequency of bacilli with associated actin tails. As shown in Fig. 5B and C, all three CCB treatments significantly reduced the frequency of actin polymerization in both *R. parkeri* and *R. conorii*-infected endothelial cells. Together, these data demonstrate that CCBs universally inhibit rickettsial actin-mediated motility.

## DISCUSSION

This study investigated the intersection of obligate intracellular *Rickettsia* pathogens and the host Ca^2+^ system with the goals of (i) exploring the potential of CCBs as novel anti-infectives, (ii) advancing the understanding of *Rickettsia* intracellular pathogenesis, and (iii) clarifying the cell biology of the mammalian Ca^2+^ system. Our investigation demonstrated that an intact Ca^2+^ system is required for maximal *Rickettsia* infection of endothelial cells, and that dysregulation of this system impedes rickettsial actin-mediated motility.

The use of CCBs effectively inhibited *Rickettsia* intracellular growth at concentrations that were not toxic to the host endothelial cells (Fig. 1), which provides grounds for their potential application toward Rickettsiosis treatment. CCBs are commonly prescribed medications that have been routinely used to reduce hypertension (54, 80) and act by impeding the influx of Ca^2+^ through various voltage-gated calcium channels to relax smooth/cardiac muscle cells (54). Since both CCBs and *Rickettsia* act primarily on the vasculature, CCBs could be considered as therapeutic candidates for *Rickettsia* infection (81–85). In addition, the only approved anti-*Rickettsia* medication in the USA is doxycycline, but the persistent 5%–10% Rickettsiosis fatality is largely attributed to doxycycline failure (5, 6, 9). Since *Rickettsia* are resistant to the vast majority of antibiotics, the concerns for *Rickettsia* developing potential resistance against doxycycline are high (9, 35, 37–39). Targeting host machinery to restrict *Rickettsia* growth using non-antibiotic medications would also reduce the risk of developing bacterial resistance (43).

Conversely, several factors need to be taken into account when considering the potential application of CCBs as anti-*Rickettsia* therapeutics. First, a major clinical hallmark of rickettsioses is the hyperpermeability of the vascular endothelial lining with systemic blood vessel leakage and hypotension (9). CCBs are designed to lower blood pressure. This fact increases concern for the feasibility of using such medication for an infection linked to hypovolemia. Second, our analysis with three distinct classes of CCBs demonstrated that all CCBs possess anti-*Rickettsia* properties but vary in their capability of affecting the *Rickettsia* intracellular life cycle. While all CCBs act on L-type calcium channels, Bepridil is a non-selective CCB that also acts on not only other types of voltage-gated Ca^2+^ channels (T-type) but also other non-calcium channels such as ATP-sensitive K^+^ channels and Na^+^-activated K^+^ and Na^+^ channels (86–90). The Bepridil TCIC_50_ is approximately two times the routinely achieved human serum levels. Verapamil is capable of inhibiting L-, T-, N-, P-, and Q-type voltage-gated calcium channels (91–93), as well as K^+^ channels and the efflux membrane protein P-glycoprotein 1 (93–95). Despite anti-*Rickettsia* activity, Verapamil is not a good candidate for therapeutic use since treatment of *R. conorii* infected endothelial cells induced host cell apoptosis (Fig. 4). Nisoldipine and Benidipine (96) are both dihydropyridines, which are the most common class of CCBs that target voltage-gated calcium channels for which the *Rickettsia* TCIC_50_ is within the normally achieved human serum concentration (71). Despite having a delaying effect on endothelial hyperpermeability *in vitro*, administration of Benidipine into *R. parkeri*-infected C3H/HeN mice only delayed hyperpermeability in the liver, but the mice experienced exacerbated clinical symptoms and increased mortality (96). Consequently, CCB candidates and their off-target effects should be carefully assessed. Finally, one should consider the ethical factors of using CCBs as potential therapeutics alone. Rickettsioses remain lethal diseases; therefore, studies should be performed to determine if CCBs have synergistic effects with the current frontline (doxycycline) treatment (97).

Our investigation highlighted that *Rickettsia* infection results in an influx of Ca^2+^ into the cytoplasm (Fig. 2). Eukaryotic cells maintain a cytoplasmic Ca^2+^ concentration that is much lower than the corresponding extracellular concentration (47, 48). Cytoplasmic Ca^2+^ ions are pumped into the extracellular space by Ca^2+^-ATPases and Na^+^/Ca^2+^ exchangers, into the sarco-/endoplasmic reticulum by SERCA enzymes, into lysosomes via the lysosome Ca^2+^ importer LCI (98), or into mitochondria by the MCU complex and VDAC proteins (99). Conversely, Ca^2+^ ions can rapidly flux back into the cytoplasm via voltage-gated or receptor-gated plasma membrane calcium channels on the plasma membrane, ryanodine or IP_3_ receptors on the ER membrane, and TRPML1/TPC on lysosomal membranes (47). A pressing question from our investigation is, what is the physiological mechanism of the rapid and persistent influx of Ca^2+^ ions into the cytoplasm after *Rickettsia* infection? Herein, we demonstrate (i) rapid calcium influx/depolarization after *Rickettsia* invasion but before the bacteria would be able to translate and secrete effector proteins (Fig. 2D) and, (ii) a persistent elevation of intracytoplasmic Ca^2+^ (Fig. 2A). The initial (<40 minute) depolarization can be temporally linked to two *Rickettsia* activities, invasion into the host cell and phagolysosomal escape. First, *Rickettsia* invasion requires host signaling proteins that also intersect with Ca^2+^-associated cell signaling, including PI3K, PIP5K, PI(4,5)P2, Cdc42, c-src, Arf6, c-Cbl, and receptor tyrosine kinases (2, 17, 19, 100). Namely, *Rickettsia* invasion depends on the phosphoinositide kinase/phosphatase network and the soluble product IP_3_, which are also the primary activators of receptor-operated ER calcium channels (101, 102). Second, rickettsial escape from the nascent phagolysosome is another potential source of calcium ion flux through direct phospholipase activities or release of lysosomal calcium stores. *Rickettsia* escape from phagosomes through secreted phospholipase enzymes. While rickettsial enzymes that promote escape from the phagosome have phospholipase A2 and D activities (103–109), the bacteria also possess poorly defined phospholipase C activity that would result in the production of the calcium channel activator IP_3_ (110, 111). In addition, early lysosomal fusion would release lysosomal Ca^2+^ stores into the nascent *Rickettsia*-containing phagosome, which would be subsequently released into the cytoplasm after *Rickettsia*-mediated lysis (112). Together, these potential linkages between early *Rickettsia* pathogenesis and the rapid increase in cytoplasmic Ca^2+^ require further investigation.

One obvious candidate for the later (12 hour post-infection) source of cytoplasmic Ca^2+^ would be the plasma membrane voltage-gated transporters, as chemical inhibition of these transporters with CCBs inhibits *Rickettsia* proliferation (Fig. 1). Endothelial cells lack the dramatically fluctuating membrane potential commonly associated with neurons but still possess a substantial resting potential of −40 to −70 mV, voltage control of cell membrane Ca^2+^ channels, and intercellular voltage potentiation across gap junctions (113, 114). In addition, a recent report highlighted extensive *R. parker*i*/*endoplasmic reticulum (ER) contact (115). These physical interactions have significant potential to release the significant ER calcium stores into the cytoplasm. Together, these two major sources of Ca^2+^ ions likely contribute to the persistent elevation of cytoplasmic Ca during intracellular *Rickettsia* infection. This persistent elevation of intracellular Ca^2+^ would also have substantial effects on various endothelial cell functions, including prostacyclin production, nitric oxide production, oxidative stress, vascular permeability, activation of secreted matrix metalloproteases, and communication with the underlying vascular smooth muscle cells (99, 101, 116, 117).

Our analysis of CCB influence on rickettsial adherence/invasion (Fig. 3) and modulation of host apoptosis (Fig. 4) revealed that CCBs have minor and inconsistent effects on these pathogenic processes. We attribute these nominal variations to the broad effects of disrupting the host cell calcium equilibrium, while not directly toxic to the host cells (Fig. 1 and 2), may influence eukaryotic cell biology. With regard to host cell apoptosis, multiple inputs, intracellular signaling, and physiological changes can ultimately result in changes to apoptosis (118, 119). Within the Rickettsiales, the intraphagosomal pathogen *Anaplasma phagocytophilum* has the most clearly defined interaction with host cell apoptosis, whereby the pathogen drastically delays neutrophil apoptosis through mitochondria membrane potential, caspase 3, JAK/STAT, and AFAP molecular pathways (118, 120–130). Conversely, pathogens in the genus *Rickettsia* have a more nuanced relationship with host cell apoptosis, whereby *R. rickettsii* inhibits endothelial and arthropod cell apoptosis through a caspase-3/NF-κB dependent mechanism (28–30, 75, 131, 132), but *R. parkeri* appears to induce mitochondria-dependent apoptosis in tick cells (76, 133). Data from Fig. 4 and Fig. S1 demonstrate that different *Rickettsia* species may possess variable inherent apoptosis phenotypes, so the relationship between *Rickettsia* and apoptosis may be more nuanced than a universal pro- or anti-apoptosis interaction that requires further considerations like *Rickettsia* species/strain, host cell, and temporal variables.

The strongest and most consistent phenotype that we observed was CCB-mediated disruption of *Rickettsia* actin polymerization (Fig. 5). At this point, we cannot conclude that there is a direct linear connection between host cell calcium channel inhibition and rickettsial actin polymerization, as disruption of Ca^2+^ homeostasis is detrimental to the host cell and thus could be expected to have a negative effect on *Rickettsia*. Additionally, these findings are only partially aligned with previous findings that genetic ablation of rickettsial actin polymerization disrupts intercellular spread and virulence but not intracellular proliferation (26). Actin is a major component of the cellular cytoskeletal structure and is known to play crucial roles in structural maintenance, phagocytosis, inflammasome assembly, and autophagy (27, 134–136). The processes employed by *Rickettsia* spp. to manipulate actin occur in two distinct phases. (i) Early polymerization by RickA to form short “comet”-like actin tails through the host Arp2/3 complex, similar to *Shigella* and *Listeria* (77, 78, 137). However, our data implicates the Ca^2+^ system in rickettsial polymerization later in the infection in the cytoplasm (Fig. 3). (ii) Later in infection, Sca2 is responsible for the characteristic long filamentous actin tails polymerized by SFG *Rickettsia* during replication (Fig. 5) (26, 78, 79). Transposon deletion of Sca2 in *Rickettsia* resulted in smaller plaque size and milder clinical symptoms in guinea pig models without affecting the bacteria’s intracellular replication (26). Our finding that CCBs restrict *Rickettsia* actin polymerization and that this phenotype is linked to growth inhibition supports the concept that actin is important for *Rickettsia* virulence.

Spotted Fever group *Rickettsia* are not unique in their ability to polymerize host actin (27, 45, 134, 138). Therefore, it is reasonable to ask if there is a broad relationship between bacterial actin polymerization and the Ca^2+^ system. *Shigella flexneri* infection induces global changes in [Ca^2+^]i, and localized InsP3-mediated intracellular Ca^2+^ signaling is crucial for bacterial invasion (57, 139, 140). For *Listeria monocytogenes*, treatment with non-specific calcium channel antagonists or calcium-free media both reduce bacterial entry significantly by influencing the pore-forming protein Listerialysin O (141). In our case, some CCB treatments marginally affect *Rickettsia* cell adherence but have no effect on bacterial invasion (Fig. 3). Listerial Ca^2+^ binding to the actin-severing protein gelsolin has been implicated to effectively cap the budding end to keep the tails short and branched (142, 143). Our data integrates into the current concept that multiple different bacteria manipulate actin in a Ca^2+^-dependent manner.

In Fig. 5, we observed that CCB treatment drastically reduces rickettsial actin polymerization. A subsequent question is how modulation of [Ca^2+^]i prevents *Rickettsia* actin assembly? Current literature suggests Ca^2+^ influences bacterial actin assembly indirectly through Ca^2+^-dependent actin binding proteins, Ca^2+^-mediated kinases, or small GTPases (144–147). Many host actin-binding proteins that directly interact with Ca^2+^, such as α-Actinin and Fibrin, are also involved in *Rickettsia* actin polymerization, suggesting possible involvement (25, 148–151). Rho GTPases such as Cdc42 and Ca^2+^-dependent kinases such as PKC have also been implicated in the process of *Rickettsia* intracellular lifecycle, indicating their potential role in the regulation of the host cytoskeleton through Ca^2+^-induced protein kinases or Rho GTPases activity (147). Thus, it would be worthwhile to define the molecular mechanisms of the tripartite *Rickettsia*/Ca^2+^/actin interaction to further define the complex process of *Rickettsia*-mammalian interaction.

The host Ca^2+^ signaling system has been demonstrated to influence other pathogens closely related to *Rickettsia*. Within the order Rickettsiales, Verapamil-mediated inhibition of Ca^2+^ influx interferes with internalization and intramonocytic replication of *Ehrlichia chaffeensis* and *Ehrlichia canis* (61–63). Also, *Orientia tsutsugamushi* is known to activate Ca^2+^ signaling pathways through phospholipase C in non-phagocytic mammalian cells and prevent excessive intracellular Ca^2+^ release upon induced apoptosis to prevent cell death (64, 65). Taken together, these findings demonstrate that many rickettsial pathogens manipulate the host Ca^2+^ system to enhance pathogenesis.

CCBs have also been shown to possess novel inhibitory effects on other more distally related Gram-negative intracellular bacteria (*Coxiella burnetii*, *Legionella pneumophila*, and *Brucella abortus*) during the initial chemical screen of FDA-approved compounds, implying that CCBs could have novel therapeutic potential across different obligate intracellular Gram-negative bacteria (40, 62, 63), including *Chlamydia *spp*. *(152–154), *Listeria monocytogenes* (44, 55, 155), *Mycobacterium tuberculosis* (156–159), and *Salmonella enterica* (160–162). These findings implicate the potential of CCBs for being a novel therapeutic against intracellular bacterial infections, while also providing evidence that host cell Ca^2+^ plays important roles in the intracellular lifecycle of many bacterial pathogens. The next step of this investigation would be to pinpoint the host elements involved in our established Ca^2+^-*Rickettsia* infection phenotype by using advanced sequencing techniques and analysis. Our lack of understanding of how Ca^2+^ is regulated throughout these interactions may help fill in the pieces of the puzzle that are left in the understanding of mammalian calcium regulation.

Altogether, our data revealed that *Rickettsia* infection of endothelial cells results in and requires potent influxes of Ca^2+^ into the host cell cytoplasm. Using various CCBs to disrupt host Ca^2+^ systems effectively restricted *Rickettsia* growth through inhibiting rickettsial polymerization of host actin via yet-to-be-characterized mechanisms. Overall, this analysis showed that mammalian host Ca^2+^ systems are an integral part of *Rickettsia* pathogenesis, and CCBs have therapeutic potential against *Rickettsia* infections.

## MATERIALS AND METHODS

### Mammalian cells

Mammalian cells were routinely maintained at 37°C and 5% CO_2_. Vero cells (17) and EA.hy926 cells (68) were cultured in DMEM (Gibco) supplemented with 10% heat-inactivated fetal bovine serum (FBS, Atlanta Biologicals), HyClone MEM non-essential amino acid solution (Thermo Fisher), and L-Glutamine (Gibco). For assays involving Ca^2+^-free media, EA.hy926 cells were grown to confluency before replacing the media with Ca^2+^-free media consisting of MEM Eagle Joklik’s Formulation (JMEM, Lonza), HyClone MEM non-essential amino acid solution, sodium pyruvate, 20% dialyzed FBS (Gibco), and supplemented with 0.5 mM CaCl_2_ where noted.

### Bacteria

*R. parkeri* strain “Portsmouth” and *R. conorii* strain “Malish 7” were routinely propagated in Vero cells and purified by sucrose density gradient as previously described (163, 164). *Rickettsia* titers were determined by Reed and Muench’s limiting dilution assay (20). MOI values were carefully chosen to reflect the goals of each experiment.

### Drug treatment

Confluent monolayers of EA.hy926 cells in 96-well plates were treated with noted concentrations of Bepridil hydrochloride (Torcis), Verapamil hydrochloride (Thermo), or Nisoldipine (Thermo) solubilized in DMSO (final concentration 1% DMSO) for 30 minutes prior to infection. *R. conorii* was added at an MOI of 3, and contact with the host cell was induced by centrifugation at 200 × *g*. Mock (DMSO)-treated/*Rickettsia-*infected and mock (DMSO)-treated/uninfected wells were used as controls for 100% and 0% *R*. *conorii* growth, respectively. All infections were incubated for 3 days at 37°C and 5% CO_2_ prior to fixation and 0.1% Triton X-100 permeabilization. Samples were then labeled with rabbit anti-*Rickettsia* polyclonal antibody RcPFA (165) and anti-rabbit IgG-Alexafluor488 (Invitrogen). Fluorescence was measured at 488 nm excitation and 530 nm emission with a 515 nm cutoff in a SpectraMax M2 plate reader (Molecular Devices). To calculate 0% *Rickettsia*-mediated fluorescence, the average fluorescence of mock (DMSO)-treated/uninfected wells was subtracted from all wells. To determine % *R. conorii* growth, all drug-treated *R*. conorii-infected samples were divided by the average of the mock-treated/*R. conorii*-infected samples and multiplied by 100. All drug-treated samples were graphed, and the best fit was generated by Nonlinear fit of [drug] vs normalized response-variable slope (GraphPad Prism). Fifty percent tissue culture inhibitory concentrations were determined from these best-fit values.

Confluent monolayers of EA.hy926 cells were treated similarly to assess toxicity. Mock-treated (0.1% DMSO) and a fatal concentration of 10% DMSO were used as 0% toxic and 100% toxic treatments, respectively. After 3 days, cells were subjected to the CellQuant MTT metabolic assay (Thermofisher). To calculate 100% toxicity (i.e., 0% survival), the average of the 10% DMSO-treated (killed) wells was subtracted from all values. To determine % toxicity, all drug-treated values were divided by the average of the mock-treated values and multiplied by 100. All drug-treated samples were graphed, and the best fit was generated by Nonlinear fit of [drug] vs normalized response-variable slope (GraphPad Prism). Fifty percent toxicity concentrations were determined from these best-fit values.

### Depletion of extracellular calcium

Confluent monolayers of EA.hy926 cells were washed with calcium-free PBS and then supplied with JMEM media containing dialyzed FBS or dialyzed FBS + 0.5 mM CaCl_2_. After 30 minutes, cells were infected with *R. parkeri* at MOI = 3 and centrifuged to ensure contact. Samples were incubated for 3 days at 37°C and 5% CO_2_ to allow *Rickettsia* growth with minimal cell viability differences. Infected samples were fixed, permeabilized, and stained with RcPFA and anti-rabbit IgG-AlexaFluor488 for fluorescent plate reader readings. MTT cell viability assays were performed as described above to assess cell viability.

### Analysis of intracellular calcium concentrations

Confluent monolayers of EA.hy926 were washed with appropriate buffer (166), and loaded for 30 minutes with 5 µM Fluo-4 AM ester (Molecular Probes) with an equal volume of 20% wt/vol Pluronic F-127 at room temperature. After incubation, the cells were incubated in 1× HEPES buffered saline for 30 minutes at 37°C to allow de-esterification of Fluo-4. Background Fluo-4 fluorescence was established for 6 minutes by reading every 10 seconds at 37°C at 490 nm excitation and 525 nm emission with a 515 nm cutoff. MOI = 0, 10, 20, 40, or 80 *R*. *parkeri* were added and contact induced by centrifugation at 200 × *g*. Plates were then immediately transferred back to the plate reader to read for an additional 40 minutes. Relative fluorescence intensity was calculated by subtracting the average of baseline fluorescence values from each fluorescence reading. This value was subsequently divided by the average baseline fluorescence and multiplied by 100 ($\Delta F/F=\frac{F_{timepoint}-F_{average\;baseline}}{F_{average\;baseline}}\times 100$). Differences in relative fluorescence between time points were quantified by subtracting the relative fluorescence at t = 40 minutes from the relative fluorescence at t = 0. Data are mean and SEM of 12 biological replicates, and an unpaired *t*-test was performed where noted. For analysis 12 hours post-infection, EA.hy926 cells were infected with MOI = 10 *R*. *parkeri* for 12 hours before being loaded with Fluo-4 as described in the previous section.

### Visualization of intracellular calcium

EA.hy926 cells were loaded with Fluo-4 for 30 minutes prior to or 12 hours post*-R. parkeri* MOI = 10 infection, respectively. CellTracker Blue (Invitrogen) was added as a control. Live cells were analyzed using a DeltaVision Elite deconvolution microscope at 40×. Micrographs were analyzed by measuring the mean pixel intensity of the whole image in ImageJ. Selected background coordinates were the same for each Fluo-4 and CellTracker picture set. Videos were acquired live using the LSM800 confocal microscope.

### Adherence and invasion

To assess bacterial adherence and invasion, EA.hy926 cells were treated with 0.1% DMSO, 10 µM Bepridil, 50 µM Verapamil, 10 µM Nisoldipine, or 1 µM Cytochalasin D for 30 minutes prior to infection. Cells were stained and analyzed as performed previously (19). Bacteria and nuclei were quantified manually using ImageJ. Cell association was determined by dividing the total amount of *Rickettsia* by the number of nuclei in at least 25 fields. % Invasion was determined by dividing the number of intracellular *Rickettsia* by the number of total *Rickettsia*.

### Analysis of apoptosis

Confluent monolayers of EA.hy926 cells in 6-well plates were infected with *R. parkeri* or *R. conorii* at MOI = 3 for 24 hours prior to drug treatment. As a positive control for apoptosis, infected cells were heat shocked at 55°C for 20 minutes. Annexin-V PE/7-AAD FACS kit (BD Scientific) was used to label all samples as per the manufacturer’s instructions. After liberation by trypsin, cells infected with *R. parkeri* were stained before loading; cells infected with *R. conorii* were stained for 15 minutes, washed, fixed with 4% paraformaldehyde, and subjected to flow cytometric analysis. Data were analyzed using FlowJo. To visualize apoptosis, cells were stained with DAPI and mounted on slides for imaging using an LSM800 confocal microscope at 40×.

### Analysis of actin polymerization

Confluent monolayers of EA.hy926 cells grown on glass cover slips were infected with MOI = 3 *R*. *parkeri* or *R. conorii,* with contact induced by centrifugation. After 24 hours of incubation, cells were treated with noted concentrations of drugs. After an additional 24 hours, cells were fixed with paraformaldehyde and permeabilized. Cells were then stained with anti-*Rickettsia* RcPFA, anti-rabbit IgG-Aleafluor488, and Phalloidin-Alexafluor555. The presence of actin tails was determined by manual counting of at least 60 cells.

## SUPPLEMENTAL MATERIAL

Fig. S1 iai.00363-25-s0001.docx *R. parkeri* Portsmouth induces host cell death in EA.hy926 cells.

ASM does not own the copyrights to Supplemental Material that may be linked to, or accessed through, an article. The authors have granted ASM a non-exclusive, world-wide license to publish the Supplemental Material files. Please contact the corresponding author directly for reuse.
