# Supplementary material for: The host calcium system contributes to intracellular Rickettsia pathogenesis
Source: Infect Immun. 2025 Aug 21;93(10):e00363-25. doi: 10.1128/iai.00363-25 (PMC12519782; doi:10.1128/iai.00363-25)
Supplement: Fig. S1 — R. parkeri Portsmouth induces host cell death in EA.hy926 cells. [file iai.00363-25-s0001.docx]

**Supplemental Material**

**
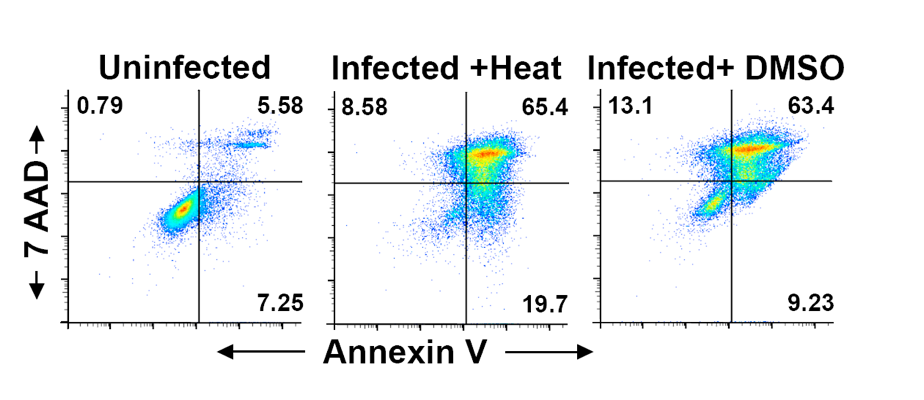
**

**Supplemental Figure 1. *R. parkeri* Portsmouth induces host cell death in EA.hy926 cells. (A)** Cells were labeled with Annexin V to detect apoptotic cells and 7-AAD to detect non-viable cells. Flow cytometric analysis of non-infected EA.hy926 cells versus 48-hour *R. parkeri* strain Portsmouth infected EA.hy926 endothelial cells after heat shock or mock (DMSO) treatment. Both heat shock and mock treatments increase the frequency of apoptotic or non-viable cells after *R. parkeri* strain Portsmouth infection indicating that these treatments induce apoptosis of infected EA.hy926 cells.
